# Individualizing Inpatient Diabetes Management During the Coronavirus Disease 2019 Pandemic

**DOI:** 10.1177/1932296820923045

**Published:** 2020-05-05

**Authors:** Francisco J. Pasquel, Guillermo E. Umpierrez

**Affiliations:** Division of Endocrinology, Metabolism, and Lipids, Emory University School of Medicine, Atlanta, GA, USA

**Keywords:** diabetes, COVID-19, inpatient, hospitalized, hyperglycemia

## Abstract

Diabetes is associated with poor clinical outcomes in hospitalized patients with coronavirus disease 2019 (COVID-19). During this pandemic, many hospitals have already become overwhelmed around the world and are rapidly entering crisis mode. While there are global efforts to boost personal protective equipment (PPE) production, many centers are improvising care strategies, including the implementation of technology to prevent healthcare workers’ exposures and reduce the waste of invaluable PPE. Not optimizing glycemic control due to clinical inertia driven by fear or lack of supplies may lead to poor outcomes in patients with diabetes and COVID-19. Individualized care strategies, novel therapeutic regimens, and the use of diabetes technology may reduce these barriers. However, systematic evaluation of these changes in care is necessary to evaluate both patient- and community-centered outcomes.

The rapidly spreading coronavirus disease 2019 (COVID-19) pandemic is having profound consequences on patients, healthcare workers (HCW), healthcare systems, and the global economy. Several healthcare systems have already become overwhelmed around the world and are rapidly entering crisis mode.^1^

Many hospitalized patients with COVID-19 have underlying chronic health conditions. In the United States, diabetes is among the most common underlying condition affecting people with severe COVID-19 and is associated with high mortality.^2,3^ Observational data from a single hospital in Wuhan, suggest patients with diabetes and severe COVID-19 have a significantly higher case-fatality rate compared to those without diabetes.^3^ Diabetes was also associated with prolonged length of stay and higher resource utilization.^3^

It is well established that inpatient hyperglycemia contributes to a significant increase in morbidity, mortality, and healthcare costs, and that better glycemic control may improve clinical outcomes.^4,5^ Insulin therapy has been considered the regimen of choice in the hospital. A standardized basal-bolus regimen is recommended for most noncritically ill patients.^5^ However, this approach is complex (multiple injections and frequent point-of-care [POC] glucose testing) and is associated with iatrogenic hypoglycemia.^4^ In the intensive care unit (ICU), insulin therapy is even less convenient, with patients needing hourly POC testing during continuous insulin infusion (CII).^5^

A critical shortage of personal protective equipment (PPE) has already developed in areas of high demand.^1^ With the current approach to manage inpatient diabetes in patients with COVID-19, or persons under investigation, there is increased exposure risk for HCW as well as a high utilization of PPE. There is an urgent need to implement effective glycemic control treatment approaches aiming at conserving the supply of PPE and reducing HCW exposure. The use of noninsulin agents in selected groups of patients, novel algorithms for hyperglycemic crises management, and the use of diabetes technology may be game changers in this setting.

## Critically Ill Patients

Insulin should be the therapy of choice for critically ill patients with COVID-19.^5,6^ The goal glycemic target range for ICU patients is between 140 and 180 mg/dL. To achieve this goal, the use of CII with hourly POC glucose monitoring is recommended.^5^ This current global public health crisis points out the significant burden of glycemic control on the healthcare system when caring for patients under isolation precautions, which is only intensified by the use of CII. The implementation of technology that minimizes HCW exposures while maintaining high-level care is essential.

## Hyperglycemic Crises

Patients with type 1 diabetes and acute infections are likely to develop diabetic ketoacidosis (DKA). Although less frequent, DKA may occur in type 2 diabetes during acute illness or among those with long-standing disease and precipitating factors. Older patients may be prone to hyperglycemic hyperosmolar state (HHS). Patients with mild to moderate DKA may be effectively treated with subcutaneous insulin every two to four hours; however for those with severe DKA, HHS, or combined DKA-HHS, CII is recommended. Our current approach with hourly POC glucose testing is clearly impractical. There is an urgent need to systematically learn novel approaches utilizing diabetes technology (ie, continuous glucose monitoring [CGM] and artificial pancreas).

## Noncritically Ill Patients

A basal-bolus approach includes the administration of basal insulin (half of total daily dose [TDD]) plus rapid-acting insulin before meals (half of TDD divided into three) plus supplemental insulin before meals and at bedtime. This complex regimen demands multiple POC fingersticks and injections throughout the day. The role of this approach is reasonable for patients with significantly uncontrolled glucose levels or type 1 diabetes; however, it may lead to overtreatment in patients with type 2 diabetes with mild hyperglycemia (BG <180 mg/dL).^4^

**Figure 1.**
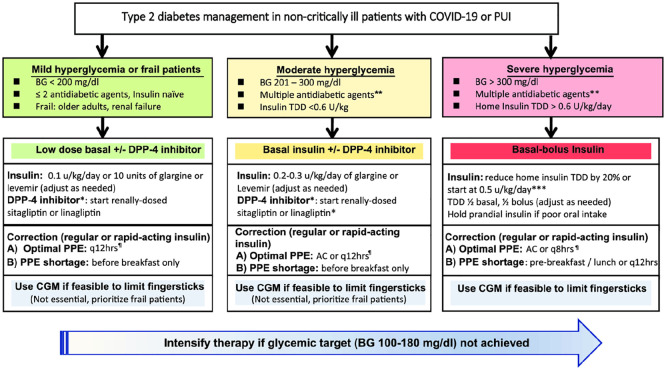
Individualized antihyperglycemic therapy in in non-critically ill patients with type 2 diabetes during the Covid-19 pandemic. AC, before meals; BG, blood glucose; CGM continuous glucose monitoring; Covid-19, coronavirus disease 2019; DPP-4, dipeptidyl peptidase-4; GLP1, glucagon-like peptide 1; PPE, personal protective equipment; PUI, persons under investigation; TDD, total daily dose. *Consider Saxagliptin if no renal failure or congestive heart failure. **Antidiabetic agents: oral agents and/or GLP1-RA. ***In patients with hypoglycemia risk (frail: elderly, renal failure) reduce starting dose to 0.15 U/kg/day (basal alone) or TDD 0.3 U/kg/day (basal bolus). ^¶^Monitor glucose levels once a day if stable glycemic control is achieved for more than two days, intensify if change in clinical status. No prospective studies have determined the efficacy of other oral antidiabetic drugs in the hospital setting. Metformin is commonly used in the hospital (renally dosed metformin is associated with low risk of lactic acidosis). The use of premixed insulin regimens is discouraged in the hospital. Intravenous insulin therapy is the therapy of choice in critically ill patients. Adapted from Pasquel et al.^4^ with permission from Springer Nature (Copyright 2019).

Despite limited evidence, oral agents are commonly used in the hospital.^4^ Dipeptidyl peptidase-4 (DPP-4) inhibitors have been studied in randomized trials. These agents, with known cardiovascular safety, are associated with: (a) simplification of therapy (fewer insulin injections), (b) proven efficacy compared to more complex regimens, and (c) good tolerability.^4,6^ It should be clear, however, that these agents seem to be mostly effective in patients with mild to moderate hyperglycemia^4^ (Figure 1). The combination of a DPP-4 inhibitor with basal insulin was as effective as basal-bolus insulin in non-ICU patients (home TDD < 0.6 units/kg/day). This regimen was associated with low risk of hypoglycemia, less insulin use, and a reduction in the number of injections.^6^ DPP-4 inhibitors carry a small increase in risk for nasopharyngitis, which should be monitored. Results of large clinical trials have not revealed any clinically relevant safety concerns related to infections, immune, or inflammatory disorders.^6^ DPP-4 inhibition could have a theoretical role in Middle East Respiratory Syndrome (MERS), as DPP-4 (also known as CD26) serves as the port of entry for MERS-CoV. The clinical relevance of this is not known in COVID-19, given SARS-CoV-2, which binds to angiotensin-converting enzyme 2.

Glucagon-like peptide (GLP)-1 receptor agonists along with basal insulin are also attractive but may increase the risk of gastrointestinal side effects. More research is needed with weekly GLP-1 receptor agonists during acute illness.^4^ Other oral agents such as thiazolidinediones (slow onset of action, volume retention), sulfonylureas (hypoglycemia risk), or sodium-glucose cotransporter-2 inhibitors (euglycemic DKA, genitourinary infections) may be impractical during this pandemic. Metformin requires close monitoring for lactic acidosis in the presence of respiratory illness, hypoxia, and acute kidney injury.^4^

## Diabetes Technology in the Hospital

Advances in CGM and automated insulin delivery have revolutionized diabetes management. Recent efforts have shown feasibility data in the hospital setting.^8^ Studies using closed-loop insulin delivery have shown remarkable improvements in glycemic control compared with usual care in noncritically ill patients.^8^ The integration of computer-guided insulin infusion with CGM or further development of new automated insulin delivery systems in the ICU is urgently needed. If feasible and implemented appropriately, CGM with remote monitoring may be ideal during this pandemic. The U.S. Food and Drug Administration (FDA) allowed both Dexcom and Abbott to supply CGM devices for inpatient use during the current public health care crisis. In press releases in early April, both Abbot and Dexcom announced their efforts to join the fight against COVID-19.^9,10^ Inpatient clinical trials are systematically testing the accuracy, safety, and efficacy of CGM technology in the hospital (NCT03877068, NCT03508934). We hope the implementation of CGM technology across hospitals is: a) opportune; b) individualized (if limited availability); c) used to safely guide therapy under these circumstances; d) helps preserve PPE; and more importantly, e) protects our frontline healthcare workers and the community.

## Conclusion

Effective inpatient diabetes treatment approaches that can reduce the effort of medical staff resulting from multiple insulin injections and fingerstick testing, the waste of invaluable PPE, and patient discomfort during this pandemic are paramount. Not optimizing glycemic control due to clinical inertia driven by fear or lack of supplies may lead to poor outcomes in patients with diabetes and COVID-19. An individualized approach, as opposed to standardized regimens, may reduce these barriers during this pandemic. However, systematic evaluation of these changes in care is necessary to evaluate both patient- and community-centered outcomes.^11^

## References

[1] Livingston E Desai A Berkwits M . Sourcing personal protective equipment during the COVID-19 pandemic [published online ahead of print March 28, 2020]. doi:10.1001/jama.2020.5317 32221579

[2] CDC COVID-19 Response Team. Preliminary estimates of the prevalence of selected underlying health conditions among patients with coronavirus disease 2019 - United States, February 12-March 28, 2020. MMWR Morb Mortal Wkly Rep. 2020;69(13):382-386. 32240123 10.15585/mmwr.mm6913e2 PMC7119513

[3] Yan Y Yang Y Wang F , et al. Clinical characteristics and outcomes of severe COVID-19 patients with diabetes. BMJ Open Diabetes Res Care. 2020; 8(1):e001343. 10.1136/bmjdrc-2020-001343 PMC7222577 32345579

[4] Pasquel FJ Fayfman M Umpierrez GE. Debate on insulin vs non-insulin use in the hospital setting-is it time to revise the guidelines for the management of inpatient diabetes? Curr Diab Rep. 2019;19(9):65. 31353426 10.1007/s11892-019-1184-8

[5] Umpierrez GE Hellman R Korytkowski MT , et al. Management of hyperglycemia in hospitalized patients in non-critical care setting: an endocrine society clinical practice guideline. J Clin Endocrinol Metab. 2012;97(1):16-38. 22223765 10.1210/jc.2011-2098

[6] Drucker DJ. Coronavirus infections and type 2 diabetes-shared pathways with therapeutic implications. Endocrine Reviews. 2020;41(3):bnaa011. https://academic.oup.com/edrv/article/doi/10.1210/endrev/bnaa011/5820492 10.1210/endrev/bnaa011 PMC7184382 32294179

[7] Pasquel FJ Gianchandani R Rubin DJ , et al. Efficacy of sitagliptin for the hospital management of general medicine and surgery patients with type 2 diabetes (Sita-Hospital): a multicentre, prospective, open-label, non-inferiority randomised trial. Lancet Diabetes Endocrinol. 2017;5(2):125-133. 27964837 10.1016/S2213-8587(16)30402-8

[8] Davis GM Galindo RJ Migdal AL Umpierrez GE. Diabetes technology in the inpatient setting for management of hyperglycemia. Endocrinol Metab Clin North Am. 2020;49(1):79-93. 31980123 10.1016/j.ecl.2019.11.002 PMC7453786

[9] Abbott. Press release. Accessed April 9, 2020. https://abbott.mediaroom.com/2020-04-08-Abbotts-FreeStyle-R-Libre-14-Day-System-Now-Available-in-U-S-for-Hospitalized-Patients-with-Diabetes-During-COVID-19-Pandemic

[10] Dexcom. Fact Sheet for Healthcare Providers: Use of Dexcom Continuous Glucose Monitoring Systems During the COVID-19 Pandemic. Accessed April 23, 2020 https://www.dexcom.com/hospitalfacts

[11] Nacoti M Ciocca A Giupponi A , et al. At the epicenter of the Covid-19 pandemic and humanitarian crises in Italy: changing perspectives on preparation and mitigation [published online ahead of print March 21, 2020]. NEJM Catal. doi:10.1056/CAT.20.0080

